# Supplementary material for: A GPER-PKA-Centrin axis regulates centrosome numbers and centriole integrity in colon cancer cells
Source: Commun Biol. 2025 Nov 26;8:1739. doi: 10.1038/s42003-025-09249-4 (PMC12673129; doi:10.1038/s42003-025-09249-4)
Supplement: Supplementary file 2 — Supplementary Information [file 42003_2025_9249_MOESM2_ESM.pdf]

Supplementary Information

A GPER-PKA-Centrin axis regulates centrosome numbers and centriole integrity in colon cancer cells

Jeanine Fahrländer, Miriam Bühler, Julia Martins Shih, Catrin Zordick, Madleen Busse, Markus Becker, Gilbert Schönfelder, and Ailine Stolz

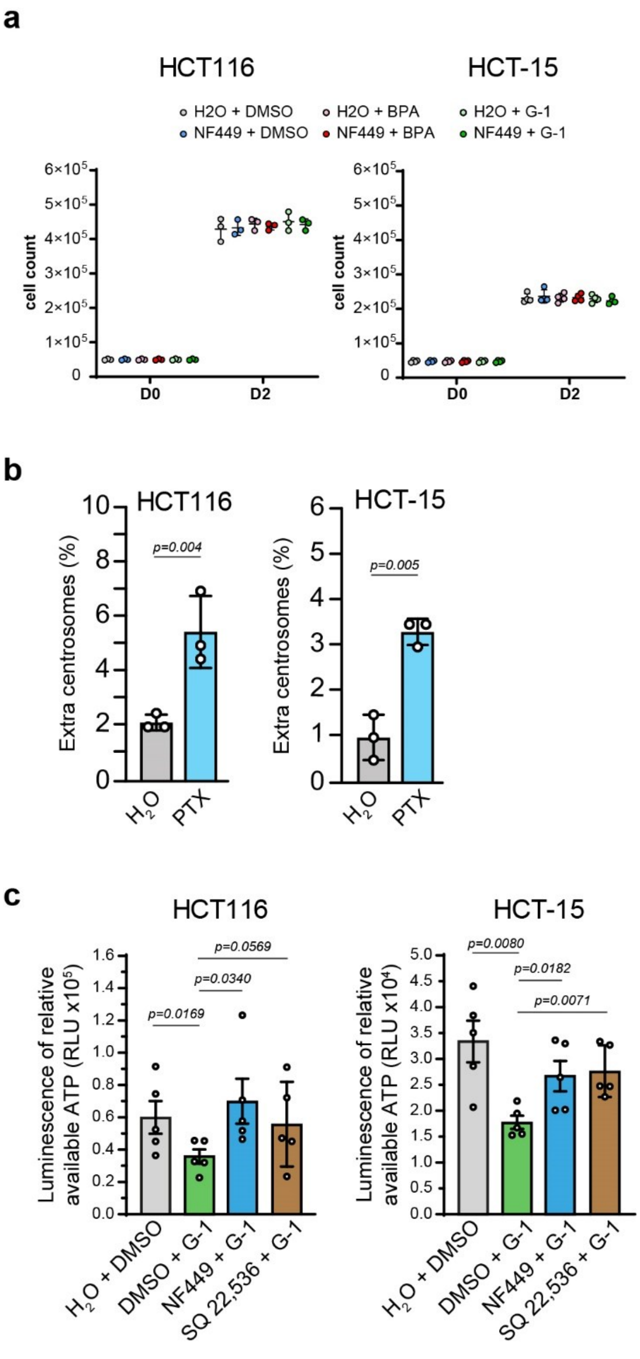

Supplementary Figure 1. Gαi or adenylyl cyclase inhibition restores GPER1-induced cAMP elevation and promotes centrosome amplification without altering proliferation. a Cells were treated with DMSO as solvent control, Bisphenol A (BPA) or G-1 to induce GPER1-activity with or without 30 min pre-treatment with NF449 to inhibit PKA. 5 x 10^4 cells were seeded per 12-well (HCT116) or 6-well plates (HCT-15) and manually quantified on day 0 and 2 using a

hemacytometer and by trypan blue exclusion of dead cells. **b** Quantification of interphase HCT116 (a) and HCT-15 cells (b) with more than two centrosomes upon treatment with Pertussis toxin (PTX) for 48 h. **c** Relative Luminescent Units (RLU) of relative available ATP upon inhibition of Gαs or adenylyl cyclase before activation of GPER1 with synthetic ligands for 48 h.

Data information: All graphs show mean and error ± SD (a, b) or mean ± SEM (c), and individual data points from three (a, HCT116) and (b), four (a, HCT-15) or five (c) different experiments with a total of 600 interphase cells (b). P values <0.05 are displayed. The following statistics were applied: *bootstrap* procedure for graphs in (b) as described in the Materials and Methods section and Paired t-test for graphs in (c).

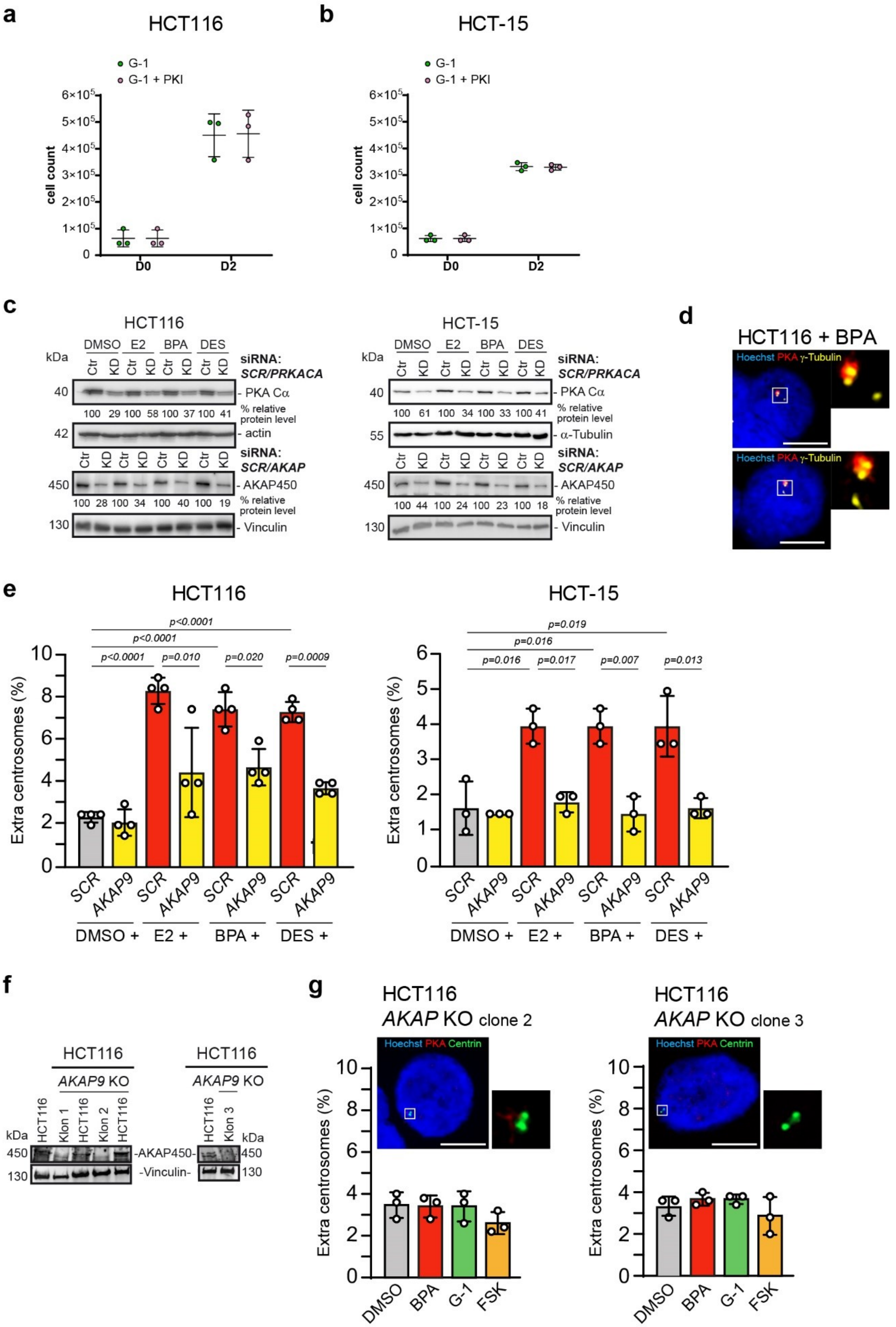
a
HCT116
G-1
G-1 + PKI
cell count
D0
D2
b
HCT-15
G-1
G-1 + PKI
cell count
D0
D2
c
HCT116
DMSO E2 BPA DES
siRNA: SCR/PRKACA
kDa
Ctr KD Ctr KD Ctr KD Ctr KD
40 - PKA Cα
100 29 100 58 100 37 100 41
% relative protein level
42 - actin
siRNA: SCR/AKAP
450 - AKAP450
100 28 100 34 100 40 100 19
% relative protein level
130 - Vinculin
HCT-15
DMSO E2 BPA DES
siRNA: SCR/PRKACA
40 - PKA Cα
100 61 100 34 100 33 100 41
% relative protein level
55 - α-Tubulin
siRNA: SCR/AKAP
450 - AKAP450
100 44 100 24 100 23 100 18
% relative protein level
130 - Vinculin
d
HCT116 + BPA
Hoechst PKA γ-Tubulin
e
HCT116
p<0.0001
p<0.0001
p<0.0001
p=0.010
p=0.020
p=0.0009
Extra centrosomes (%)
SCR AKAP9 SCR AKAP9 SCR AKAP9 SCR AKAP9
DMSO + E2 + BPA + DES +
HCT-15
p=0.019
p=0.016
p=0.016
p=0.017
p=0.007
p=0.013
Extra centrosomes (%)
SCR AKAP9 SCR AKAP9 SCR AKAP9 SCR AKAP9
DMSO + E2 + BPA + DES +
f
HCT116
AKAP9 KO
HCT116 Klon 1 HCT116 Klon 2 HCT116
-AKAP450-
-Vinculin-
HCT116
AKAP9 KO
HCT116 Klon 3
kDa
450
130
g
HCT116
AKAP KO clone 2
Hoechst PKA Centrin
Extra centrosomes (%)
DMSO BPA G-1 FSK
HCT116
AKAP KO clone 3
Hoechst PKA Centrin
Extra centrosomes (%)
DMSO BPA G-1 FSK

Supplementary Figure 2. Inhibition of PKA does not affect cell proliferation. a, b Cells were treated with GPER1-specific agonist for 48 h with or without 30 min pre-treatment with 5 µM PKI (14-22) amide to inhibit PKA. 5 x 10^4 cells were seeded per 6-well plates and manually quantified on day 0 and 2 using a hemacytometer and by trypan blue exclusion of dead cells. **c** Whole-cell lysates were immunoblotted from cells treated with DMSO or xenoestrogens as indicated. Actin, α-tubulin or Vinculin served as loading controls. Relative protein levels were normalized to the loading controls and expressed relative to *SCRAMBLED* siRNA (Ctr, control). KD, Knockdown; SCR, Scrambled; AKAP, AKAP450. **d** Representative images of interphase cells treated with Bisphenol a (BPA) for 48 h and co-immunostained with anti-PKA and anti-γ-tubulin antibodies (insets), and stained with Hoechst 33342. Scale bar: 10 µm. Note the displaced Centrin-2 signals in centrosome amplified cells. **e** Quantification of interphase HCT116 (left) and HCT-15 (right) cells with more than two centrosomes upon siRNA-mediated knockdown of *AKAP9* (encoding for AKAP450) before activation of GPER1 with endogenous or xenoestrogen ligands for 48 h. **f** Whole-cell lysates were immunoblotted from HCT116 parental cells and three different cell clones derived from HCT116 cells depleted of AKAP450 (*AKAP9* knockout, KO). Vinculin served as a loading control. KO, knockout. **g** Quantification of interphase cells with more than two centrosomes upon activation of GPER1 with a xenoestrogen ligand (BPA) or synthetic agonist (G-1) or PKA with forskolin (FSK) for 48 h in HCT116 *AKAP450* knockout cell clones (*AKAP* KO, clone 1 and clone 3). Representative images of AKAP450-depleted cells were shown. Cells were co-immunostained with anti-PKA and anti-Centrin antibodies (insets), and stained with Hoechst 33342. Scale bar: 10 µm. Note the absence of PKA in AKAP450-depleted cells.

Data information: All graphs show mean and error ± SD and individual data points from three (a, b; e, HCT-15, and g) or four (e, HCT116) different experiments with a total of 600 (HCT-15) or 800 interphase cells (HCT116), or 1500 interphase cells (i). P values <0.05 are displayed. The following statistics were applied: Paired t-test for graphs in (a) and (b) and *bootstrap* procedure for graphs in (e and g), as described in the Materials and Methods section.

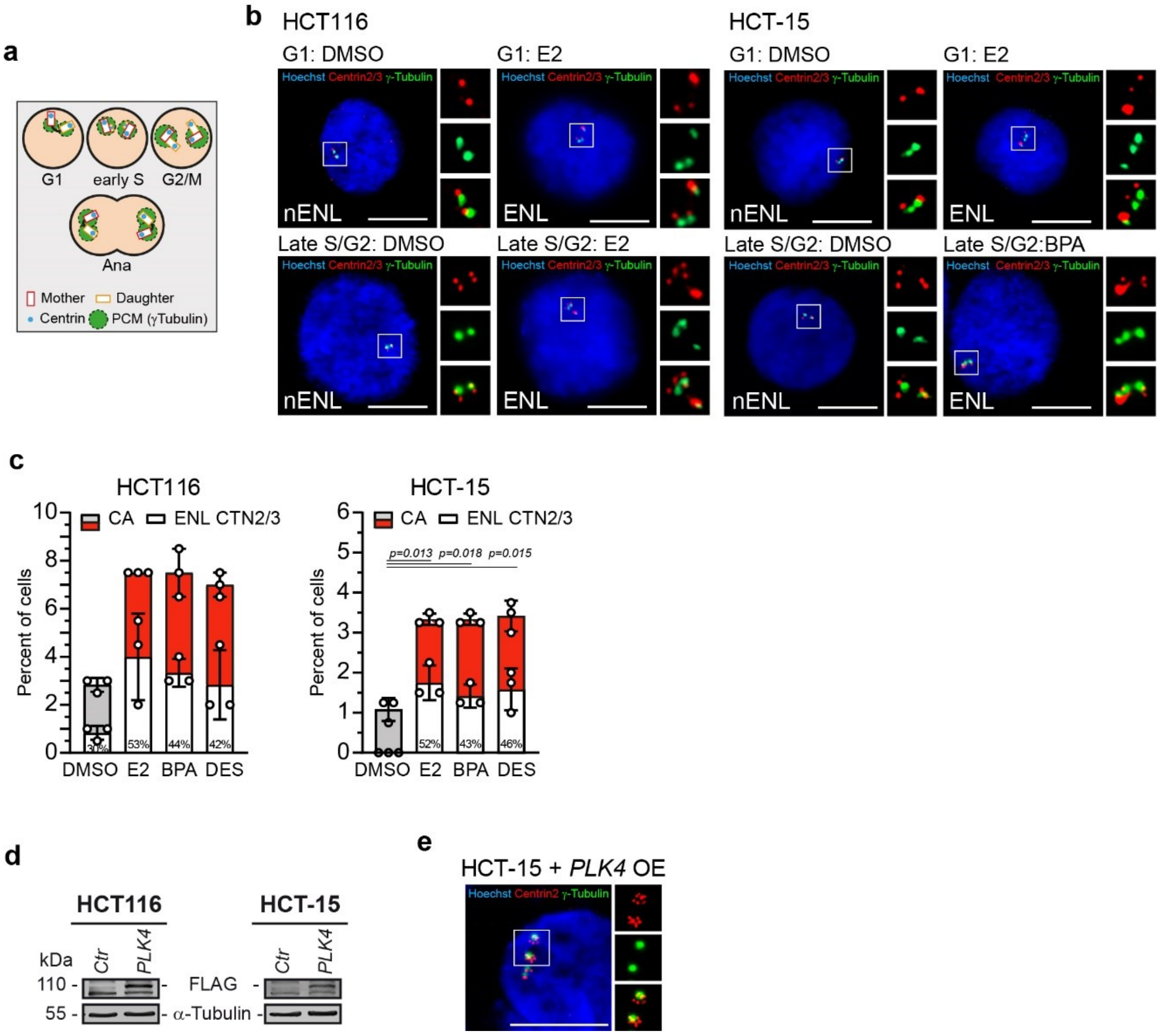

Supplementary Figure 3. Activation of GPER1 leads to enlarged centriolar Centrin2/3 but other centriole markers are not affected. a Schematic of centriole numbers throughout the eucaryotic cell cycle. **b** Representative images of interphase cells upon activation of GPER1 with endogenous or xenoestrogen ligands for 48 h, with or without enlarged Centrin-2 *foci* (Insets) at G1 or late S/G2 phase. Scale bar: 10 µm. **c** Cells were treated with enogoenous or xenoestrogen GPER1 ligands for 48 h to induce GPER1 mediated centrosome amplification. The bar graphs show quantification of interphase cells with more than two centrosomes (CA) and the proportion of centrosome amplified cells with enlarged Centrin-2/3 *foci* (Enlarged, ENL). Percentages of enlarged Centrin-2/3 *foci* are given. **d** Whole-cell lysates were immunoblotted from cells transfected with empty vector (Control, Ctr) or *PLK4* (pCMV-flag-*PLK4*). α-tubulin served as a loading control. **e** Representative image of an interphase cell expressing *PLK4* with amplified centrosomes (Insets). Scale bar: 10 µm.

Data information: All graphs in (d) show mean ± SD and individual data points from three different experiments with a total of 1200 amplified interphase cells. P-values for enlarged Centrin-2 <0.05 are displayed. P-values for centrosome amplification are given in the

Supplementary Table 1. The following statistics were applied: *bootstrap* procedure for graphs in (d) as described in the Materials and Methods section.

a

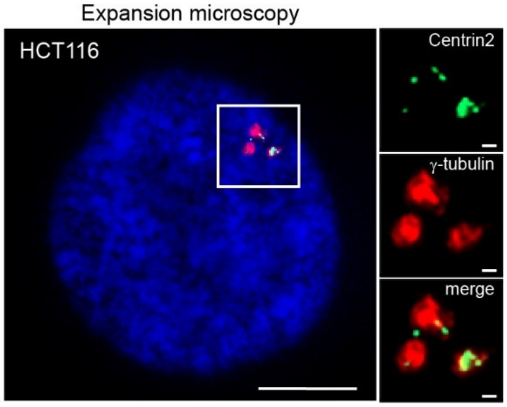

b

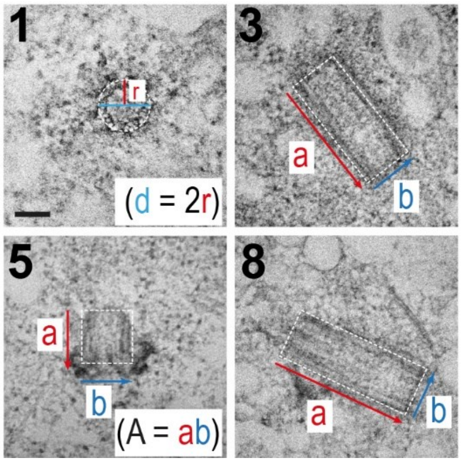

c

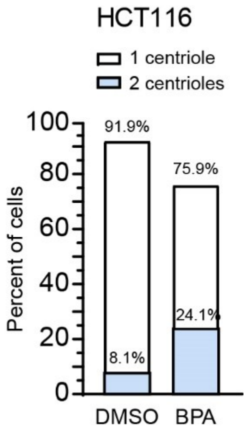

Supplementary Figure 4. Activation of GPER1 causes aberrant centrioles with enlarged Centrin *foci* at amplified centrosomes. a, Representative expansion microscopy image of cells treated with 10 nM BPA with multiple Centrin *foci* assembled into a super spot. Scale bar: 10 μm. Expansion factor of 3.5x. Scale bar of insets: 2 μm. **b** Representative transmission electron microscopy images of HCT116 cells, as shown in Fig. 4e, illustrating the measurements of centriole radius (d) and cross-sectional area (A). **c** HCT116 cells were treated with DMSO (solvent control) or Bisphenol A (BPA) for 48 h to induce GPER1-mediated centrosome amplification. The proportion of cells with one or two centrioles per TEM-image was determined. **Data information:** The graphs in (c) shows a barr diagram with a total of 37 (DMSO) and 58 (BPA) cells. The number (n) of cells evaluated for the proportion of cells with one or two centrioles per image are as followed: $n_{(DMSO)}$ = 34, $n_{(BPA)}$ = 44 cells with one centriole, and $n_{(DMSO)}$ = 3, $n_{(BPA)}$ = 14 cells with two centrioles per image.

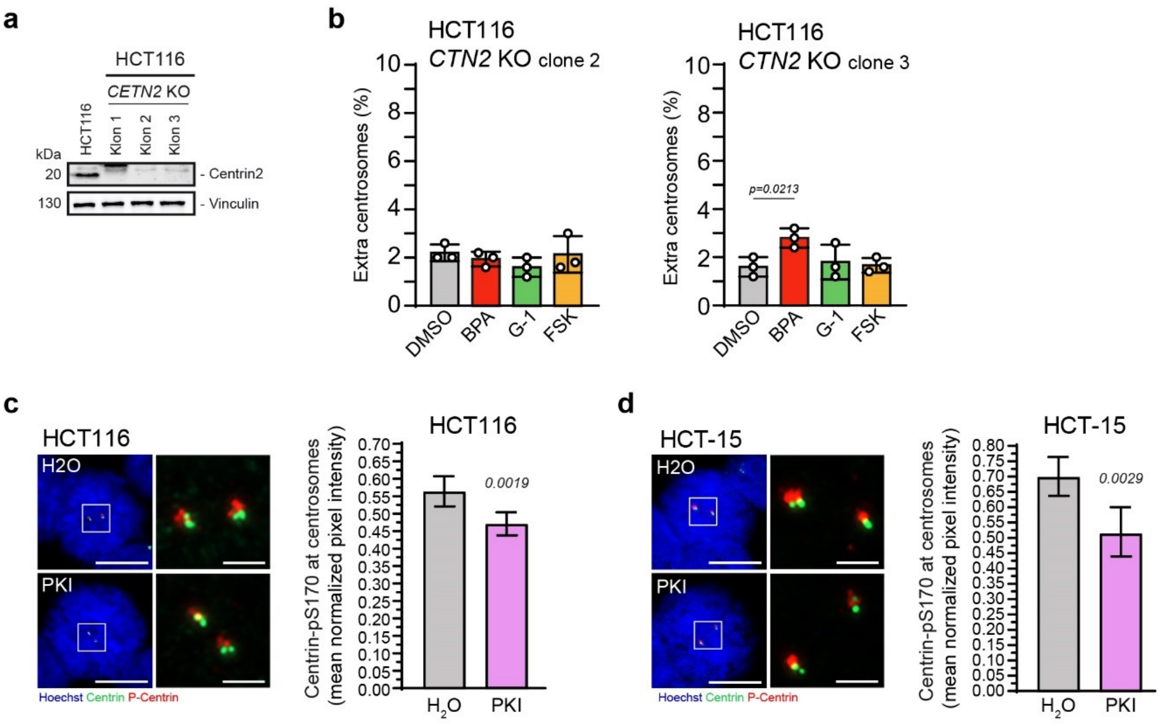

Supplementary Figure 5. Inhibition of PKA reduces phospho-Centrin-2 at mitotic centrosomes. a Whole-cell lysates were immunoblotted from HCT116 parental cells and three different cell clones derived from HCT116 cells depleted of Centrin-2 (*CTN2* knockout, KO). Vinculin served as a loading control. KO, knockout. **b** Quantification of HCT116 interphase cell clones depleted of Centrin-2 (*CTN2* knockout, KO) with more than two centrosomes upon activation of GPER1 and PKA for 48 h. **c, d** Representative images of prometaphase cells upon inhibition of PKA for 48 h and co-immunostained with anti-Centrin-2-pS170 and anti-Centrin antibodies and stained with Hoechst3342. Scale bars: 10 µm. Insets show magnified Centrin-2 and P-Centrin-2 signals (scale bar, 2 µm). The bar graphs show fluorescence intensities of P-Centrin-2 normalized to signals for total Centrin-2 from prometaphase cells treated with H2O (solvent) or PKI (14-22) amide for 48 h ($n_{(HCT116,\ DMSO)}$ = 43; $n_{(HCT116,\ PKI)}$ = 43; $n_{(HCT\text{-}15,\ DMSO)}$ = 44; $n_{(HCT\text{-}15,\ PKI)}$ = 44 prometaphase cells).

Data information: Graphs in (b) show mean ± SD and individual data points from three different experiments with a total of 1500 amplified cells. Graphs in (c) show geometric mean ± 95% CI from three independent experiments. P-values <0.05 are displayed. The following statistics were applied: *bootstrap* procedure for graphs in (b) and Mann–Whitney's test for graphs in (c).

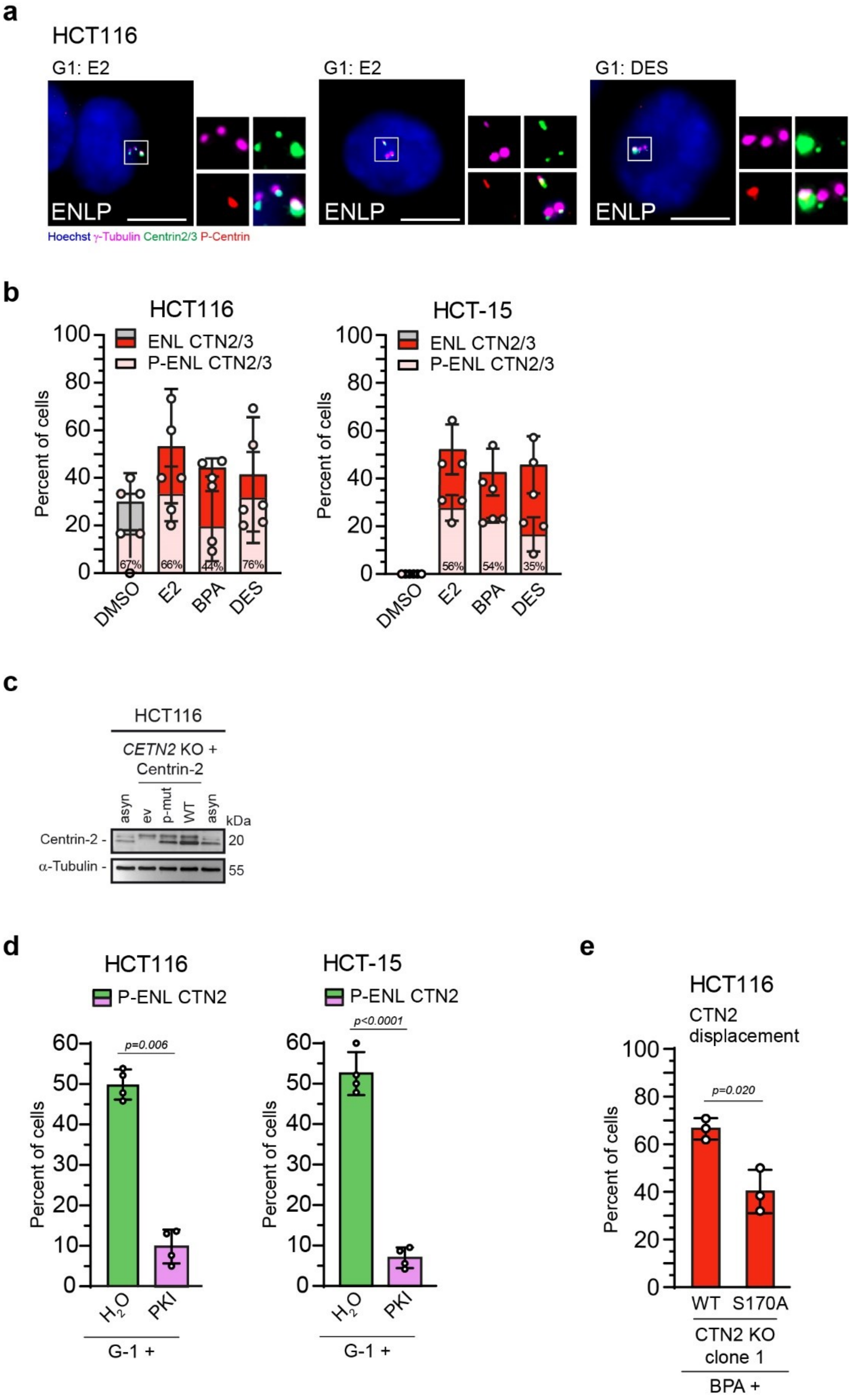
a
HCT116
G1: E2
G1: E2
G1: DES
ENLP
ENLP
ENLP
Hoechst γ-Tubulin Centrin2/3 P-Centrin
b
HCT116
HCT-15
ENL CTN2/3
P-ENL CTN2/3
Percent of cells
67%
66%
44%
76%
56%
54%
35%
DMSO
E2
BPA
DES
c
HCT116
CETN2 KO +
Centrin-2
asyn
ev
p-mut
WT
asyn
kDa
Centrin-2 -
α-Tubulin -
20
55
d
HCT116
HCT-15
P-ENL CTN2
p=0.006
p<0.0001
Percent of cells
H2O
PKI
G-1 +
e
HCT116
CTN2
displacement
p=0.020
Percent of cells
WT
S170A
CTN2 KO
clone 1
BPA +

Supplementary Figure 6. Enlarged centriolar Centrin-2/3 is phosphorylated at amplified interphase centrosomes upon GPER1 activation. a Representative images of interphase cells upon activation of GPER1 with enogenous or xenoestrogen ligands for 48 h with enlarged and concomitantly phosphorylated Centrin-2/3 *foci* (Insets) at G1 phase. Scale bar: 10 µm. **b** Interphase cells from Supplementary Fig. 3d upon activation of GPER1 with endogenous or xenoestrogen ligands for 48 h. The bar graphs show quantification of amplified interphase cells with enlarged Centrin-2/3 foci (Enlarged, ENL) and the proportion of enlarged Centrin-2 phosphorylated at Ser170 (P-ENL). Percentages of enlarged and concomitantly phosphorylated Centrin-2/3 foci are given. The number (n) of cells evaluated for the proportion of phosphorylated Centrin-2 from centrosome amplified cells with enlarged Centrin-2/3 are as followed: (b) $n_{(DMSO)}$ = 4/5; $n_{(E2)}$ = 15/24; $n_{(BPA)}$ = 14/20; $n_{(DES)}$ = 17/17; (c) $n_{(DMSO)}$ = 0/0; $n_{(E2)}$ = 11/21; $n_{(BPA)}$ = 9/17; $n_{(DES)}$ = 7/19. **c** Whole-cell lysates were immunoblotted from HCT116 parental cells and HCT116 cells depleted of Centrin-2 (*CTN2* knockout, KO) re-expressing either an empty vector (ev, control), a wild-type Centrin (WT) or a PKA-non-phosphorylatable Centrin mutant (S170A, p-mut). α-tubulin served as a loading control. KO, knockout. **d** HCT116 and HCT-15 cells were treated with a synthetic GPER1 ligand for 43 h to induce GPER1-mediated centrosome amplification, followed by concurrent inhibition of PKA using PKI (14-22) amide (PKI) during the final 5 h. The bar graphs show quantification of interphase cells with phosphorylated Centrin-2 at enlarged Centrin-2 *foci* (Enlarged, ENL) upon 48 h of treatment. **e** HCT116 cells depleted of Centrin-2 (*CTN2* knockout, KO) and re-expressing either wild-type Centrin (WT) or a PKA-non-phosphorylatable Centrin mutant (S170A) were treated with Bisphenol A (BPA) for 48 h to induce GPER1-mediated centrosome amplification. The bar graphs show physically separated (i.e., displaced) amplified centrosomes.

Data information: Graphs in (b), (d), and (e) show mean ± SD and individual data points from three (b and e) or four (d) different experiments with a total of 1200 (b) or 200 (d) amplified cells. The number (n) of cells evaluated for the proportion of displaced centrosomes with enlarged Centrin-2 in (e) are as followed: $n_{(WT)}$ = 44/66; $n_{(S170A)}$ = 30/75. P-values for centrosome amplification shown in (b) are given in the Supplementary Table 1. P-values for enlarged Centrin-2/3 are given in the related Supplementary Fig. 3d. P-values for phosphorylated, enlarged Centrin-2 cannot be provided due to sample sizes.

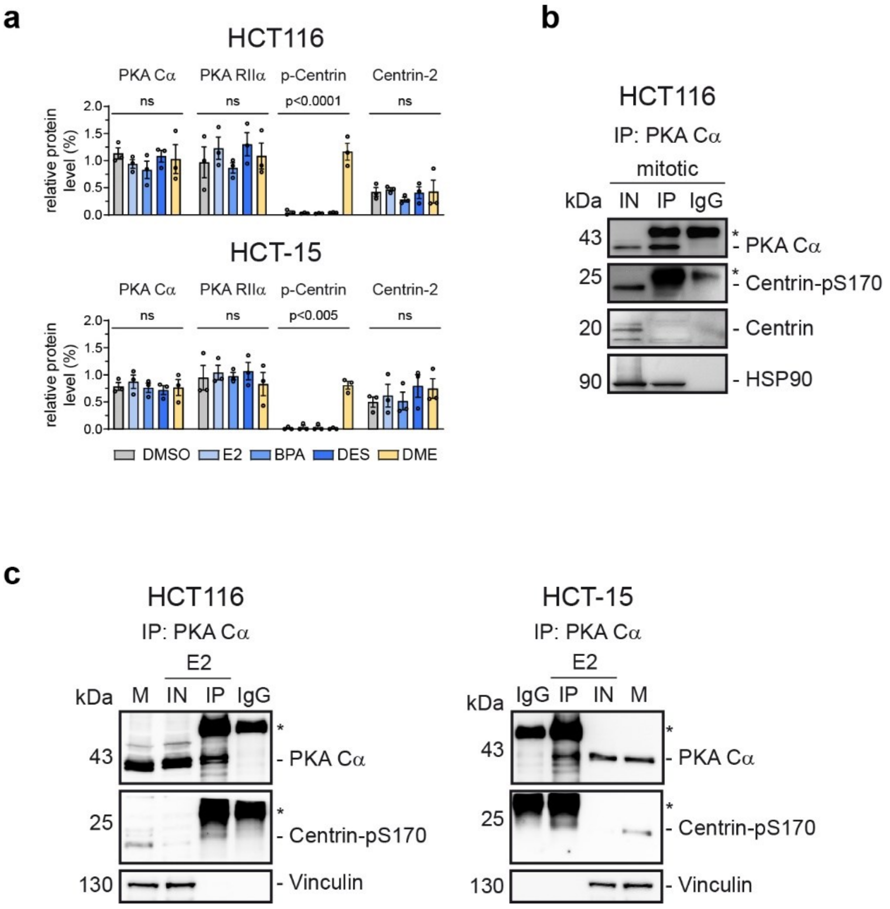

Supplementary Figure 7. GPER1 has no impact on total protein levels. a Quantification of relative protein levels normalized to Vinculin. **b** Cells were treated with 2 µM dimethylenstaron (DME) for 16 h (mitotic). Cell lysates were immunoprecipitated with anti-PKA Cα antibody. HSP90 served as a control for PKA-Cα binding (Li et al., 2021). **c** Cells were treated with 10 nM 17β-Estradiol (E2) to activate GPER1 for 48 h. Cell lysates were immunoprecipitated with anti-PKA Cα antibody. Vinculin served as a loading control.

Data information: Graphs in (a) show mean ± SEM from three different experiments. P-values <0.05 are displayed. The following statistics were applied: Two-way ANOVA with post hoc Tukey's multiple comparisons test. IP = immunoprecipitates; IN = input control (50 µg of lysate); IgG = lysate with beads and antibodies. Asterisks (*) mark unspecific or light/heavy chains of the IP capture antibody.

Original Blots

Supplementary Fig. S8. Original Westerns of Figure 5

Figure 5d

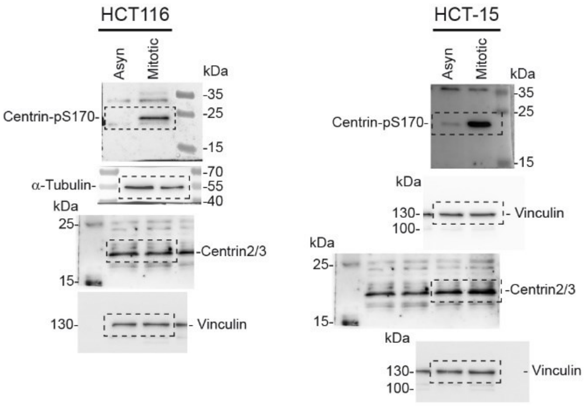

Figure 5e

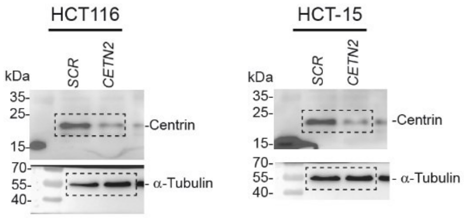

Figure 5f

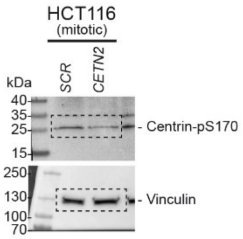

Figure 5g

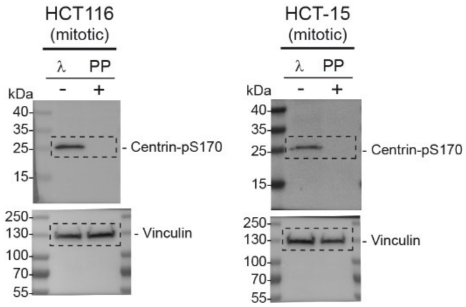

Figure 5h

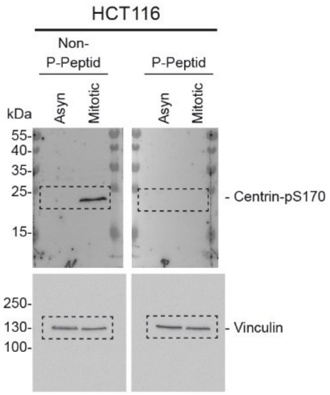

Supplementary Fig. S9. Original Westerns of Figure 7

Figure 7a

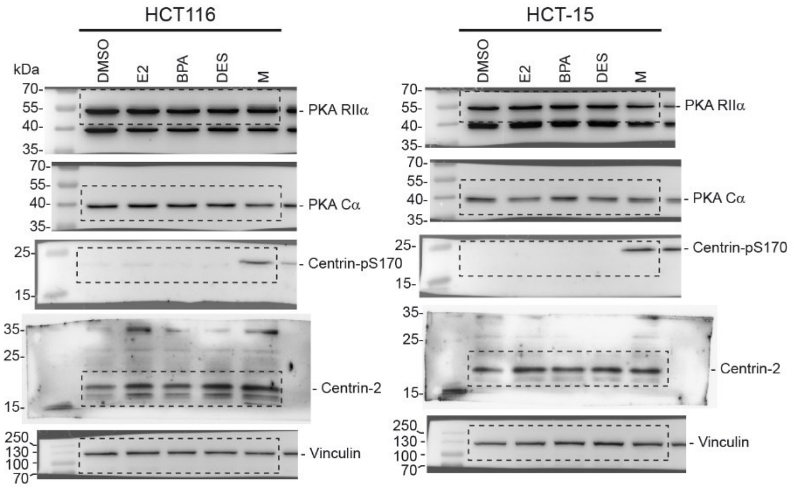

Figure 7b

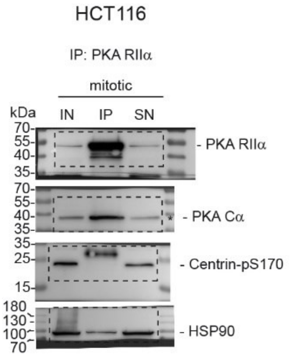

Figure 7c

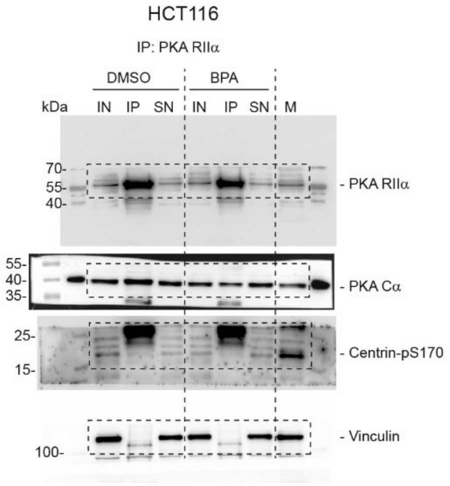

Figure 7d

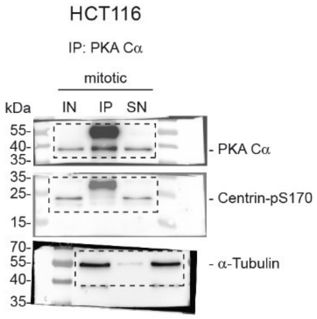

Figure 7e

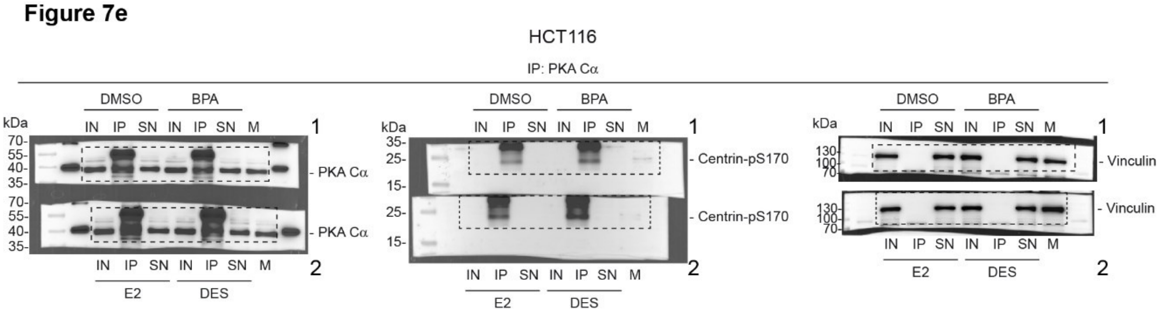

Identical numbers indicate samples run on the same gel/membrane.
Membranes 1 and 2 were blotted and developed simultaneously in the same device.

Figure 7f

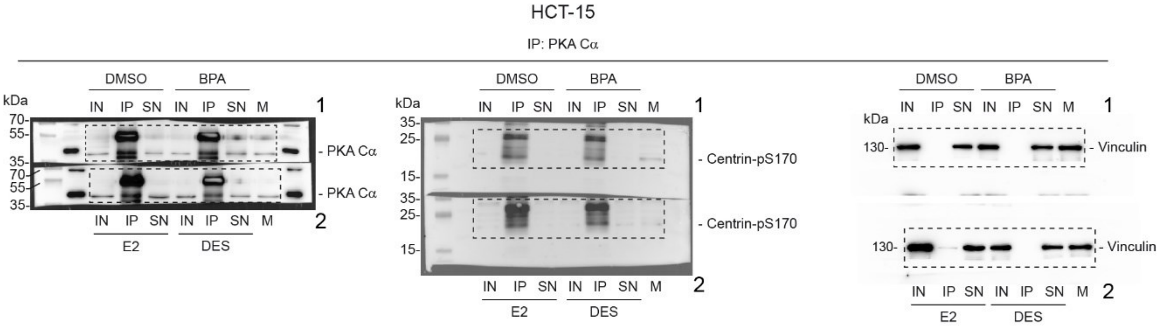

Identical numbers indicate samples run on the same gel/membrane.
Membranes 1 and 2 were blotted and developed simultaneously in the same device.

Figure 7h

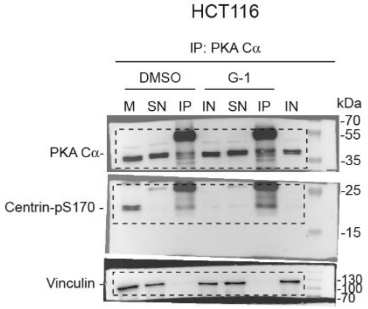

Figure 7i

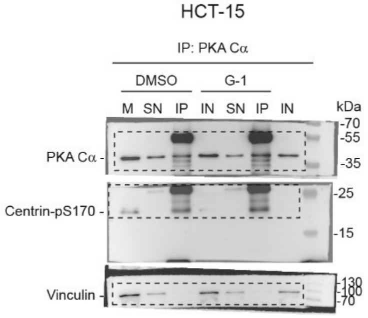

Supplementary Fig. S10. Original Westerns of Supplementary Figures

Figure S2c

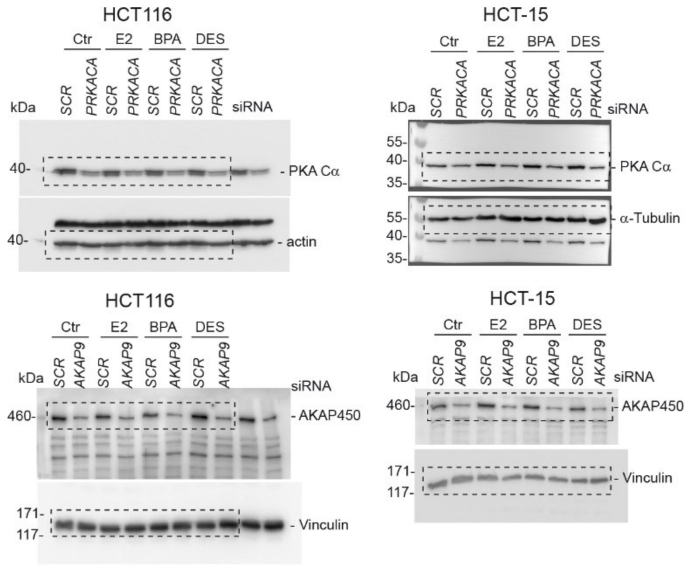

Figure S2f

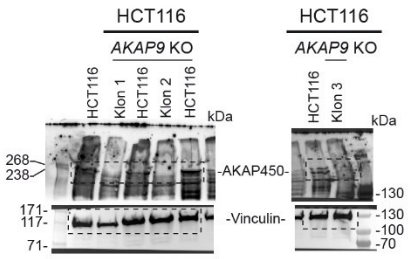

Figure S3d

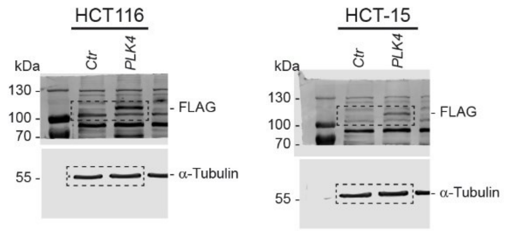

Figure S5a

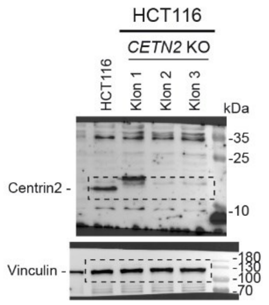

Figure S6c

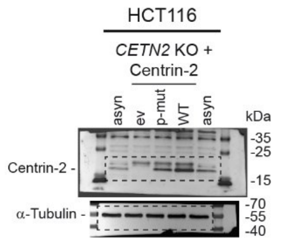

Figure S7b

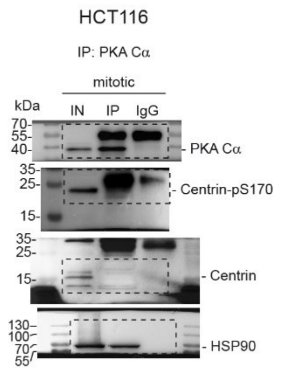

Figure S7c

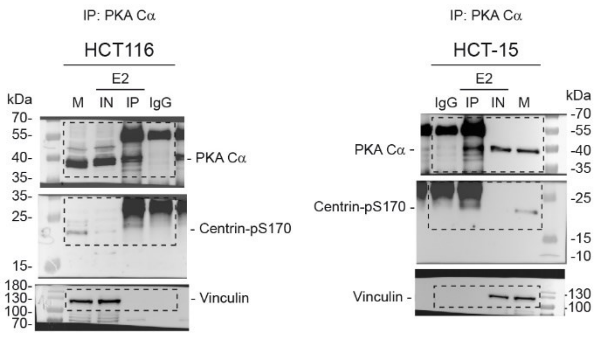

Supplementary Table 1: P-values and applied statistical test

Figure	Panel	Cell line	Treatment	p-value	Method	Assay
1	a	HCT116	DMSO		bootstrap	Centrosome amplification
			NF449	5.28e-01		
			E2	8.00e-05		
			E2 + NF449	4.29e-03		
			BPA	6.00e-05		
			BPA + NF449	7.10e-04		
			DES	1.10e-04		
			DES + NF449	3.44e-03		
1	b	HCT-15	DMSO		bootstrap	Centrosome amplification
			NF449	3.56e-01		
			E2	7.50e-03		
			E2 + NF449	1.03e-03		
			BPA	3.25e-02		
			BPA + NF449	3.23e-02		
			DES	3.29e-02		
			DES + NF449	1.67e-02		
1	c	HCT116	DMSO		bootstrap	Centrosome amplification
			NF449	3.24e-01		
			G-1	1.50e-04		
			G-1 + NF449	4.40e-04		
1	c	HCT116	DMSO		bootstrap	Centrosome amplification
			NF449	1.00e+00		
			ICI	1.90e-04		
			ICI + NF449	4.30e-04		
			Tam	1.10e-04		
			Tam+ NF449	6.30e-04		
1	d	HCT-15	DMSO		bootstrap	Centrosome amplification
			NF449	5.60e-01		
			G-1	1.27e-03		
			G-1 + NF449	1.39e-02		
1	d	HCT-15	DMSO		bootstrap	Centrosome amplification
			NF449	3.51e-01		
			ICI	1.03e-02		
			ICI + NF449	9.95e-03		
			Tam	8.44e-03		
			Tam+ NF449	2.44e-02		
1	e	HCT116	DMSO		Paired t-test (two-tailed)	cAMP Assay
			FSK	9.30e-03		
			E2	6.10e-03		
			BPA	1.48e-02		
			DES	1.82e-02		

			G-1	4.20e-03		
1	f	HCT-15	DMSO		Paired t-test (two-tailed)	cAMP Assay
			FSK	2.00e-03		
			E2	1.28e-02		
			BPA	1.15e-02		
			DES	1.27e-02		
			G-1	2.80e-02		
1	g	HCT116	DMSO		bootstrap	Centrosome amplification
			FSK	3.00e-04		
			cAMP	3.00e-05		
1	h	HCT-15	DMSO		bootstrap	Centrosome amplification
			FSK	3.87e-03		
			cAMP	6.90e-04		
1	i	HCT116	DMSO		bootstrap	Centrosome amplification
			SQ	3.04e-01		
			BPA	2.00e-05		
			E2+SQ	4.00e-04		
			BPA+SQ	9.30e-04		
			DES+SQ	1.00e-04		
1	j	HCT116	DMSO		bootstrap	Centrosome amplification
			SQ	3.28e-01		
			G-1	8.00e-05		
			G-1+SQ	3.10e-04		
1	k	HCT-15	DMSO		bootstrap	Centrosome amplification
			SQ	5.50e-01		
			BPA	3.86e-03		
			E2+SQ	4.08e-03		
			BPA+SQ	5.64e-03		
			DES+SQ	2.68e-03		
1	l	HCT-15	DMSO		bootstrap	Centrosome amplification
			SQ	5.52e-01		
			G-1	9.70e-04		
			G-1+SQ	5.73e-03		
S1	b	HCT116	DMSO		bootstrap	Centrosome amplification
			PTX	3.58e-03		
S1	b	HCT-15	DMSO		bootstrap	Centrosome amplification
			PTX	4.89e-03		

S1	c	HCT116	H_2O + DMSO		Paired t-test (two-tailed)	cAMP Assay
			DMSO + G-1	0.0169		
			NF449 + G-1	0.2258		
			SQ + G-1	0.1890		
			DMSO + G-1			
			NF449 + G-1	0.0398		
			SQ + G-1	0.0569		
S1	c	HCT-15	H_2O + DMSO		Paired t-test (two-tailed)	cAMP Assay
			DMSO + G-1	0.0080		
			NF449 + G-1	0.0954		
			SQ + G-1	0.1887		
			DMSO + G-1			
			NF449 + G-1	0.0182		
			SQ + G-1	0.0071		
2	a	HCT116	Scr		bootstrap	Centrosome amplification
			PKAsi	1.00e+00		
			E2+Scr	<1.00e-05		
			E2+PKAsi	4.70e-04		
			BPA+Scr	<1.00e-05		
			BPA+PKAsi	6.20e-04		
			DES+Scr	<1.00e-05		
			DES+PKAsi	3.78e-03		
2	b	HCT-15	Scr		bootstrap	Centrosome amplification
			PKAsi	5.50e-01		
			E2+Scr	4.56e-03		
			E2+PKAsi	6.86e-03		
			BPA+Scr	7.04e-03		
			BPA+PKAsi	6.76e-03		
			DES+Scr	5.46e-03		
			DES+PKAsi	5.32e-03		
2	c	HCT116	DMSO		bootstrap	Centrosome amplification
			PKI	1.00e+00		
			E2	2.00e-05		
			E2+PKI	4.81e-03		
			BPA	<1.00e-05		
			BPA+PKI	3.90e-04		
			DES	2.00e-05		
			DES+PKI	3.60e-04		
2	c	HCT116	DMSO		bootstrap	Centrosome amplification
			PKI	1.00e+00		
			G-1	<1.00e-05		
			G1+PKI	3.10e-04		

2	d	HCT-15	DMSO		bootstrap	Centrosome amplification
			PKI	5.45e-01		
			E2	2.28e-03		
			E2+PKI	4.46e-03		
			BPA	8.98e-03		
			BPA+PKI	4.54e-02		
			DES	1.92e-02		
			DES+PKI	2.37e-02		
2	d	HCT-15	DMSO		bootstrap	Centrosome amplification
			PKI	3.37e-01		
			G-1	8.87e-03		
			G-1+PKI	4.42e-02		
2	e	HCT116	DMSO		Paired t-test (two-tailed)	PKA activity assay
			FSK	7.00e-03		
			E2	3.10e-03		
			BPA	2.30e-03		
			DES	4.70e-03		
			G-1	<1.00e-04		
			BPA+PKI	3.70e-01		
2	f	HCT-15	DMSO		Paired t-test (two-tailed)	PKA activity assay
			FSK	3.19e-02		
			E2	1.92e-02		
			BPA	1.77e-02		
			DES	7.30e-03		
			G-1	2.50e-03		
			BPA+PKI	7.42e-01		
2	g	HCT116	DMSO		Mann–Whitney’s test	PKA intensities
			BPA	<0.0001		
			G-1	<0.0001		
2	i	HCT116 AKAP KO clone 1	DMSO		bootstrap	Centrosome amplification
			BPA	0.6390		
			G-1	0.3009		
			FSK	0.6035		
S2	e	HCT116	Scr		bootstrap	Centrosome amplification
			AKAPsi	4.04e-01		
			E2+Scr	<1.00e-05		
			E2+AKAPsi	9.52e-03		

			BPA+Scr	<1.00e-05		
			BPA+AKAPsi	2.01e-02		
			DES+Scr	<1.00e-05		
			DES+AKAPsi	9.30e-04		
S2	e	HCT-15	Scr		bootstrap	Centrosome amplification
			AKAPsi	4.69e-01		
			E2+Scr	1.60e-02		
			E2+AKAPsi	1.73e-02		
			BPA+Scr	1.61e-02		
			BPA+AKAPsi	6.57e-03		
			DES+Scr	1.94e-02		
			DES+AKAPsi	1.27e-02		
S2	g	HCT116 AKAP KO clone 2	DMSO		bootstrap	Centrosome amplification
			BPA	0.8934		
			G-1	0.9088		
			FSK	0.1383		
S2	g	HCT116 AKAP KO clone 3	DMSO		bootstrap	Centrosome amplification
			BPA	0.3643		
			G-1	0.3465		
			FSK	0.4837		
3	c	HCT116	Ctr		Paired t-test (two-tailed)	Centriole distances
			1	7.13e-01		
			2	1.58e-02		
			3	5.00e-04		
3	c	HCT-15	Ctr		Paired t-test (two-tailed)	Centriole distances
			1	3.42e-01		
			2	5.00e-03		
			3	1.00e-04		
3	d	HCT116	DMSO		bootstrap	Centrosome amplification
			E2	1.20e-07		
			BPA	4.80e-07		
			DES	4.80e-07		
3	d	HCT116	DMSO		Adapted bootstrap	Centrosome amplified cells with enlarged Centrin
			E2	2.37e-02		
			BPA	1.98e-02		
			DES	2.59e-02		

3	e	HCT116	DMSO		bootstrap	Centrosome amplification
			G-1	6.60e-07		
3	e	HCT116	DMSO		Adapted bootstrap	Centrosome amplified cells with enlarged Centrin
			G-1	4.09e-02		
3	f	HCT116	DMSO		bootstrap	Centrosome amplification
			ICI	4.00e-07		
			Tam	3.03e-06		
3	f	HCT116	DMSO		Adapted bootstrap	Centrosome amplified cells with enlarged Centrin
			ICI	2.85e-02		
			Tam	4.45e-02		
3	d	HCT-15	DMSO		bootstrap	Centrosome amplification
			E2	4.32e-04		
			BPA	3.77e-05		
			DES	3.05e-04		
3	d	HCT-15	DMSO		Adapted bootstrap	Centrosome amplified cells with enlarged Centrin
			E2	1.81e-02		
			BPA	1.59e-02		
			DES	1.78e-02		
3	e	HCT-15	DMSO		bootstrap	Centrosome amplification
			G1	3.65e-05		
3	e	HCT-15	DMSO		Adapted bootstrap	Centrosome amplified cells with enlarged Centrin
			G1	1.59e-02		
3	f	HCT-15	DMSO		bootstrap	Centrosome amplification
			ICI	1.25e-04		
			Tam	2.52e-04		
3	f	HCT-15	DMSO		Adapted bootstrap	Centrosome amplified cells with enlarged Centrin
			ICI	1.53e-02		
			Tam	1.45e-02		
3	g	HCT116	Ctr		bootstrap	Centrosome amplification
			PLK4 OE	2.52e-11		

3	g	HCT116	Ctr		Adapted bootstrap	Centrosome amplified cells with enlarged Centrin
			PLK4 OE	7.38e-01		
3	g	HCT-15	Ctr		bootstrap	Centrosome amplification
			PLK4 OE	<1.00e-05		
3	g	HCT-15	Ctr		Adapted bootstrap	Centrosome amplified cells with enlarged Centrin
			PLK4 OE	n.a.		
S3	c	HCT116	DMSO		bootstrap	Centrosome amplification
			E2	1.8e-04		
			BPA	2.3e-04		
			DES	5.6e-04		
S3	c	HCT116	DMSO		Adapted bootstrap	Centrosome amplified cells with enlarged Centrin
			E2	0.12245		
			BPA	0.17522		
			DES	0.27859		
S3	c	HCT-15	DMSO		bootstrap	Centrosome amplification
			E2	1.3e-04		
			BPA	1.5e-04		
			DES	1.4e-04		
S3	c	HCT-15	DMSO		Adapted bootstrap	Centrosome amplified cells with enlarged Centrin
			E2	1.3e-02		
			BPA	1.8e-02		
			DES	1.5e-02		
4	f	HCT116	DMSO		Mann–Whitney’s test	Centriole diameter
			BPA	0.1031		
4	f	HCT116	DMSO		Mann–Whitney’s test	Centriole length
			BPA	0.0006		

4	f	HCT116	DMSO		Mann–Whitney's test	Centriole width
			BPA	0.4760		
4	f	HCT116	DMSO		Mann–Whitney's test	Centriole area
			BPA	0.0107		
5	a	HCT116	Ctr		bootstrap	Centrosome amplification
			FSK	6.00e-10		
			8Br-cAMP	1.20e-07		
5	a	HCT116	Ctr		Adapted bootstrap	Centrosome amplified cells with enlarged Centrin
			FSK	2.61e-02		
			8Br-cAMP	6.14e-02		
5	a	HCT-15	Ctr		bootstrap	Centrosome amplification
			FSK	2.16e-05		
			8Br-cAMP	4.29e-05		
5	a	HCT-15	Ctr		Adapted bootstrap	Centrosome amplified cells with enlarged Centrin
			FSK	2.00e-02		
			8Br-cAMP	2.18e-02		
5	c	HCT116 CTN2 knockout clone 1	DMSO		bootstrap	Centrosome amplification
			BPA	0.7183		
			G-1	>0.9999		
			FSK	0.6877		
S5	b	HCT116 CTN2 knockout clone 2	DMSO		bootstrap	Centrosome amplification
			BPA	0.3747		
			G-1	0.1224		
			FSK	0.8991		
S5	b	HCT116 CTN2 knockout clone 3	DMSO		bootstrap	Centrosome amplification

			BPA	0.0213		
			G-1	0.7017		
			FSK	0.8305		
S5	c	HCT116	Ctr		Mann–Whitney's test	pCentrin intensities
			PKI	1.9e-03		
S5	d	HCT-15	Ctr		Mann–Whitney's test	pCentrin intensities
			FSK	2.9e-03		
6	c	HCT116	DMSO		n.a.	Phosphorylated enlarged Centrin
			E2	n.a.		
			BPA	n.a.		
			DES	n.a.		
6	c	HCT116	DMSO		Adapted bootstrap	Centrosome amplified cells with enlarged Centrin
			E2	2.37e-02		
			BPA	1.98e-02		
			DES	2.59e-02		
6	d	HCT116	DMSO		n.a.	Phosphorylated enlarged Centrin
			G-1	n.a.		
6	d	HCT116	DMSO		Adapted bootstrap	Centrosome amplified cells with enlarged Centrin
			G-1	4.09e-02		
6	d	HCT116	DMSO		n.a.	Phosphorylated enlarged Centrin
			ICI	n.a.		
			Tam	n.a.		
6	d	HCT116	DMSO		Adapted bootstrap	Centrosome amplified cells with enlarged Centrin
			ICI	2.85e-02		
			Tam	4.45e-02		
6	e	HCT-15	DMSO		n.a.	Phosphorylated enlarged Centrin
			E2	n.a.		
			BPA	n.a.		
			DES	n.a.		
6	e	HCT-15	DMSO			

			E2	1.81e-02	Adapted bootstrap	Centrosome amplified cells with enlarged Centrin
			BPA	1.59e-02		
			DES	1.78e-02		
6	f	HCT-15	DMSO		n.a.	Phosphorylated enlarged Centrin
			G-1	n.a.		
6	f	HCT-15	DMSO		Adapted bootstrap	Centrosome amplified cells with enlarged Centrin
			G-1	1.59e-02		
6	f	HCT-15	DMSO		n.a.	Phosphorylated enlarged Centrin
			ICI	n.a.		
			Tam	n.a.		
6	f	HCT-15	DMSO		Adapted bootstrap	Centrosome amplified cells with enlarged Centrin
			ICI	1.53e-02		
			Tam	1.45e-02		
6	g	HCT116	Ctr		n.a.	Phosphorylated enlarged Centrin
			FSK	n.a.		
			8Br-cAMP	n.a.		
6	g	HCT116	Ctr		Adapted bootstrap	Centrosome amplified cells with enlarged Centrin
			FSK	2.61e-02		
			8Br-cAMP	6.14e-02		
6	h	HCT-15	Ctr		n.a.	Phosphorylated enlarged Centrin
			FSK	n.a.		
			8Br-cAMP	n.a.		
6	h	HCT-15	Ctr		Adapted bootstrap	Centrosome amplified cells with enlarged Centrin
			FSK	2.00e-02		
			8Br-cAMP	2.18e-02		
6	i	HCT116	G-1 + H2O		Adapted bootstrap	Centrosome amplified cells with enlarged Centrin
			G-1 + PKI	0.1507		
6	i	HCT-15	DMSO + H2O			

			G-1 + PKI	0.1892	Adapted bootstrap	Centrosome amplified cells with enlarged Centrin
6	k	HCT116 CTN2 KO + CTN2 WT	BPA		Adapted bootstrap	Centrosome amplified cells with enlarged Centrin
		HCT116 CTN2 KO + CTN2 S170A	BPA	0.2006		
6	l	HCT116 CTN2 KO + CTN2 WT	BPA		Adapted bootstrap	Centrosome amplified cells with enlarged Centrin
		HCT116 CTN2 KO + CTN2 S170A	BPA	0.0357		
S6	b	HCT116	DMSO		n.a.	Phosphorylated enlarged Centrin
			E2	n.a.		
			BPA	n.a.		
			DES	n.a.		
S6	b	HCT116	DMSO		Adapted bootstrap	Centrosome amplified cells with enlarged Centrin
			E2	0.12245		
			BPA	0.17522		
			DES	0.27859		
S6	b	HCT-15	DMSO		n.a.	Phosphorylated enlarged Centrin
			E2	n.a.		
			BPA	n.a.		
			DES	n.a.		
S6	b	HCT-15	DMSO		Adapted bootstrap	Centrosome amplified cells with enlarged Centrin
			E2	1.3e-02		
			BPA	1.8e-02		
			DES	1.5e-02		
S6	d	HCT116	G-1 + H2O		Adapted bootstrap	Centrosome amplified cells with enlarged Centrin
			G-1 + PKI	0.0059		
S6	d	HCT-15	DMSO + H2O		Adapted bootstrap	Centrosome amplified cells

						with enlarged Centrin
			G-1 + PKI	<0.0001		
S6	e	HCT116 CTN2 KO + CTN2 WT	BPA		Adapted bootstrap	Centrin foci-displacement
		HCT116 CTN2 KO + CTN2 S170A	BPA	0.0220		
S7	a	HCT116	**PKA Cα**			
			DMSO vs. E2	0.8905	ANOVA with post hoc Tukey's multiple comparisons test	Relative protein levels
			DMSO vs. BPA	0.6168		
			DMSO vs. DES	0.9993		
			DMSO vs. DME	0.9874		
			E2 vs. BPA	0.9858		
			E2 vs. DES	0.9608		
			E2 vs. DME	0.9934		
			BPA vs. DES	0.7595		
			BPA vs. DME	0.8843		
			DES vs. DME	0.999		
			**PKA RIIα**			
			DMSO vs. E2	0.7421	ANOVA with post hoc Tukey's multiple comparisons test	Relative protein levels
			DMSO vs. BPA	0.9876		
			DMSO vs. DES	0.5261		
			DMSO vs. DME	0.9814		
			E2 vs. BPA	0.4402		
			E2 vs. DES	0.9966		
			E2 vs. DME	0.9624		
			BPA vs. DES	0.2571		
			BPA vs. DME	0.8342		
			DES vs. DME	0.8455		
			**p-Centrin**			
			DMSO vs. E2	>0,9999	ANOVA with post hoc Tukey's multiple comparisons test	Relative protein levels
			DMSO vs. BPA	>0,9999		
			DMSO vs. DES	>0,9999		
			DMSO vs. DME	<0,0001		
			E2 vs. BPA	>0,9999		
			E2 vs. DES	>0,9999		
			E2 vs. DME	<0,0001		
			BPA vs. DES	>0,9999		
			BPA vs. DME	<0,0001		
			DES vs. DME	<0,0001		
			**Centrin-2**			
			DMSO vs. E2	0.9996	ANOVA with post hoc	Relative protein levels
			DMSO vs. BPA	0.9634		
			DMSO vs. DES	>0,9999		

			DMSO vs. DME	>0,9999	Tukey's multiple comparisons test	
			E2 vs. BPA	0.9062		
			E2 vs. DES	0.9987		
			E2 vs. DME	0.9998		
			BPA vs. DES	0.9746		
			BPA vs. DME	0.9577		
			DES vs. DME	>0,9999		
S7	a	HCT-15	**PKA Cα**			
			DMSO vs. E2	0.9906	ANOVA with post hoc Tukey's multiple comparisons test	Relative protein levels
			DMSO vs. BPA	>0,9999		
			DMSO vs. DES	0.9969		
			DMSO vs. DME	>0,9999		
			E2 vs. BPA	0.9771		
			E2 vs. DES	0.9290		
			E2 vs. DME	0.9800		
			BPA vs. DES	0.9995		
			BPA vs. DME	>0,9999		
			DES vs. DME	0.9992		
			**PKA RIIα**			
			DMSO vs. E2	0.9872	ANOVA with post hoc Tukey's multiple comparisons test	Relative protein levels
			DMSO vs. BPA	0.9999		
			DMSO vs. DES	0.9676		
			DMSO vs. DME	0.9726		
			E2 vs. BPA	0.9967		
			E2 vs. DES	>0,9999		
			E2 vs. DME	0.8014		
			BPA vs. DES	0.9877		
			BPA vs. DME	0.9409		
			DES vs. DME	0.7226		
			**p-Centrin**			
			DMSO vs. E2	>0,9999	ANOVA with post hoc Tukey's multiple comparisons test	Relative protein levels
			DMSO vs. BPA	>0,9999		
			DMSO vs. DES	>0,9999		
			DMSO vs. DME	0.0015		
			E2 vs. BPA	>0,9999		
			E2 vs. DES	>0,9999		
			E2 vs. DME	0.0021		
			BPA vs. DES	>0,9999		
			BPA vs. DME	0.0020		
			DES vs. DME	0.0014		
			**Centrin-2**			
			DMSO vs. E2	0.9710	ANOVA with post hoc Tukey's multiple comparisons test	Relative protein levels
			DMSO vs. BPA	>0,9999		
			DMSO vs. DES	0.5364		
			DMSO vs. DME	0.6900		
			E2 vs. BPA	0.9852		
			E2 vs. DES	0.8831		
			E2 vs. DME	0.9587		
			BPA vs. DES	0.6016		

			BPA vs. DME	0.7510		
			DES vs. DME	0.9991		

Table S1: Exact p-values and the related statistical test are shown for each cell treatment of a given Figure. 17β-estradiol (E2), bisphenol A (BPA), diethylstilbestrol (DES), ICI182,780 (ICI), tamoxifen (Tam), Forskolin (FSK), SQ 22,536 (SQ), 8-Bromo-cAMP (8Br-cAMP), Pertussis toxin (PTX), PKI (14-22) amide (PKI), dimethylenastron (DME), Control (Ctr), Scrambled (Scr), siRNA (si), Overexpression (OE).

Supplementary Table 2: Reagents and Tools Table

Reagents/Resource	Reference or Source	Identifier or catalog number
**Antibodies**		
mouse anti-flag, clone M2	Sigma-Aldrich	Cat#: F3165; RRID:AB_259529
mouse anti-α-tubulin, clone B-5-1-2	Santa Cruz	Cat#: sc-23948; RRID:AB_628410
mouse anti-beta-actin, clone AC-15	Sigma-Aldrich	Cat#: F3022; RRID:AB_476970
mouse anti-HSP90 alpha/beta, clone F-8	Santa Cruz	Cat#: sc-13119; RRID:AB_675659
mouse anti-PKA Cα, clone A-2	Santa Cruz	Cat#: sc-28315; RRID:AB_628136
rabbit anti-PKA Cα	Cell Signaling	Cat#: 4782; RRID:AB_2170170
mouse anti-PKA IIα reg, clone H-12	Santa Cruz	Cat#: sc-137220; RRID:AB_2268608
rabbit anti-PKA IIα reg	ThermoFisher	Cat#: A301-670A; RRID:AB_1211499
rabbit anti-AKAP9	Abcam	Cat#: ab237752
rabbit anti-Centrin-2	Merk Millipore	Cat#: ABE480
mouse anti-Centrin-2/3, clone 20H5	Sigma-Aldrich	Cat#: 04-1624; RRID:AB_10563501
rat anti-Centrin-2, clone W16110A	BioLegend	Cat#: 698602; RRID:AB_2715794
mouse anti-Centrobin	abcam	Cat#: ab70448; RRID:AB_1268196
rabbit anti-phospho-Ser170-centrin-2	7TM Antibodies GmbH	This paper
mouse anti-Vinculin, clone 7F9	Santa Cruz	Cat#: sc-73614; RRID:AB_1131294
rabbit anti-Plk4	Proteintech	Cat#: 12952-1-AP; RRID:AB_2284150
rabbit anti-γ-tubulin	Sigma-Aldrich	Cat#: T3559; RRID:AB_477575
mouse anti-γ-tubulin, clone GTU88	Sigma-Aldrich	Cat#: T6557; RRID:AB_477584
Alexa Fluor® 647 anti-gamma tubulin, clone TU-30	Abcam	Cat#: ab191114; RRID:AB_2889219
rabbit anti-Cep135	Abcam	Cat#: ab75005; RRID:AB_1523339
rabbit anti-CP110	Abcam	Cat#: ab243696
HRP conjugated AffiniPure goat-anti mouse IgG	Jackson Laboratory	Cat#: 111-035-144; RRID:AB_2307391
HRP conjugated AffiniPure goat-anti rabbit IgG	Jackson Laboratory	Cat#: 111-035-146
IRDye® 800CW Goat anti-Mouse IgG Secondary Antibody	LICORbio	Cat#: 926-32210; RRID:AB_621842
IRDye® 680RD Goat anti-Rabbit IgG Secondary Antibody	LICORbio	Cat#: 926-68071; RRID:AB_10956166
Goat anti-Mouse IgG Alexa Fluor 488	Thermo Fisher Scientific	Cat#: A-11029; RRID:AB_2534088
Goat anti-Rabbit IgG Alexa Fluor 555	Thermo Fisher Scientific	Cat#: A-21428; RRID:AB_141784

Goat anti-Rat IgG Alexa Fluor 488	Thermo Fisher Scientific	Cat#: A-11006; RRID:AB_2534074
Goat anti-Mouse IgG Alexa Fluor 555	Thermo Fisher Scientific	Cat#: A-21424; RRID:AB_141780
**Chemicals, Enzymes and other Reagents**		
17β-estradiol (E2)	Sigma-Aldrich	Cat#: E8875-1G
bisphenol A (BPA)	Sigma-Aldrich	Cat#: 239658-50G
Diethylstilbestrol (DES)	Sigma-Aldrich	Cat#: D4628-5G
G-1	TOCRIS	Cat#: 3577
ICI 182,780 (ICI)	Sigma-Aldrich	Cat#: I4409-25MG
Tamoxifen (Tam)	Sigma-Aldrich	Cat#: T5648-1G
Forskolin (FSK)	Merck	Cat#: F6886-10MG
SQ 22,536 (SQ)	Merck	Cat#: S153-5MG
NF449	TOCRIS	Cat#: 1391/10
Pertussis toxin (PTX)	Enzo	Cat#: BML-G100-0050
8-Bromo-cAMP (8-Br cAMP)	Santa Cruz	Cat#: sc-201564
PKI (14-22) amide (PKI)	Enzo Life Science	Cat#: BML-P210-0500
3-Isobutyl-1-methylxanthin (IBMX)	Merck	Cat#: I5879-250MG
dimethylenastron	Sigma-Aldrich	Cat#: SML0905-5MG
penicillin/streptomycin	Pan Biotech	Cat#: 17-0618-01
Protease inhibitor cocktail	Merck	Cat#: 11873580001
Phosphatase inhibitor Cocktail	Merck	Cat#: 4906837001
Protein G-Sepharose 4 Fast Flow	VWR	Cat#: 17-0618-01
Interferin siRNA transfection	VWR	Cat#: 409-50
Torpedo DNA transfection Regent	Ibidi	Cat#: 60611
Albumin Fraktion V	ROTH	Cat#: T844.2
Paraformaldehyde solution	Alfa Aesar	Cat#: J19943
Glutaraldehyde	Sigma-Aldrich	Cat#: G5882-10X1ML
Glutaraldehyde (TEM)	Agar Scientific	Cat#: AGR1020
Ethanol	Carl Roth	Cat#: 5054.2
Osmium tetroxide	Electron Microscopy Scienes	Cat#: 19170
Uranyl acetate	Electron Microscopy Scienes	Cat#: 22400
Agar 100 resin	Agar Scientific	Cat#: AGR1045
DDSA	Agar Scientific	Cat#: R1053
BDMA	Agar Scientific	Cat#: AGR1062B
MNA	Agar Scientific	Cat#: AGR1083
Acrylamide 4K-Solution	AppliChem	Cat#: A1577
N,N′-Methylen-bis-acrylamid solution	Sigma-Aldrich	Cat#: M1533
Sodium Acrylate	Sigma-Aldrich	Cat#: 408220
Sodium sulphate	Merck	Cat#: 6649
Sodium hydroxide	Carl Roth	Cat#: 6771.3
Tannic acid	Thermo Fisher Scientific	Cat#: ACRO202425000
Proteinase K	Thermo Fisher	Cat#: EO0492
Dabco	ROTH	Cat#: 0718.2
Poly-L-lysin	Merck	Cat#: P1524-100MG
Lead(II) citrate	Science Services	Cat#: DM22409

Lambda Protein Phosphatase (Lambda PP)	New England Biolabs	Cat#: P0753S
**Critical commercial assays**		
cAMP-GloAssay	Promega	Cat#: V1501
PKA kinase activity kit	Enzo	Cat#: ADI-EKS-390A
Duolink® Proximity Ligation Assay	Merck	Cat#: DUO92101-1KT
**Experimental models: Cell lines**		
HCT116 (*H. sapiens*, male)	DSMZ	Cat#: ACC 581; RRID:CVCL_0291
HCT-15 (*H. sapiens*, male)	ATCC	Cat#: CCL-225; RRID:CVCL_0292
**Oligonucleotides**		
SCRAMBLED siRNA: 5´-CAUAAGCUGAGAUACUUCA-3´	Eurofins	custom made
PRKACA siRNA: 5'-GAACACACCCUGAAUGAAA-3'	Eurofins	custom made
AKAP9 siRNA: 5'-CAGGTTCGTGAATATATGGAA-3'	Eurofins	custom made
CETN2 siRNA: 5'-AAGCTCTTTGATGATGATGAA-3'	Eurofins	custom made
**Recombinant DNA**		
pcDNA 3.1	Gift from Prof. Dr. Ingrid Hoffmann (DKFZ, Heidelberg, Germany)	N/A
pCMVflag-Plk4	Gift from Prof. Dr. Ingrid Hoffmann (DKFZ, Heidelberg, Germany)	N/A
Centrin-2 Double Nickase Plasmid (h2)	Santa Cruz	sc-400867-NIC-2
AKAP 9 Double Nickase Plasmid (h2)	Santa Cruz	sc-405430-NIC-2
Centrin wild-type (NM_004344.3)	VectorBuilder	custom made
Centrin S170A vectors (NM_004344.3, codons 508-510 were changed to gcc)	VectorBuilder	custom made
**Software**		
ZEN 3.1 blue edition (immunofluorescence microscopy)	Carl Zeiss Microscopy GmbH, Germany	
ImageJ (intensities of bands of Western blots; TEM image analysis)	Wayne Rasband, National Institute of Health, USA	Version 1.53a
ITEM (TEM image acquisition and analysis)	Olympus	Version-ITEM-E-23082007
R (statistics)	R Foundation for Statistical Computing, Vienna, Austria.	Version 4.0.2 and 4.3.2
GraphPad PRISM	GraphPad Software Inc., USA	Version 10.1.2
Adobe Photoshop	Adobe, San Jose, CA, USA	Version 25.7.0
Adobe llustrator	Adobe, San Jose, CA, USA	Version 27.8
**Other**		
RPMI culture medium	Pan-Biotech	Cat#: P04-16515
Fetal Bovine Serum (FBS) South America, Charcoal Stripped	Th. Geyer GmbH & Co. KG (biowest)	Cat#: S181F-500 ml

Supplementary References

1. Li, K., P. Sun, Y. Wang, T. Gao, D. Zheng, A. Liu, and Y. Ni. 2021. Hsp90 interacts with Cdc37, is phosphorylated by PKA/PKC, and regulates Src phosphorylation in human sperm capacitation. *Andrology*. 9:185-195. 10.1111/andr.12862
