# Supplementary material for: A GPER-PKA-Centrin axis regulates centrosome numbers and centriole integrity in colon cancer cells
Source: Commun Biol. 2025 Nov 26;8:1739. doi: 10.1038/s42003-025-09249-4 (PMC12673129; doi:10.1038/s42003-025-09249-4)
Supplement: Supplementary file 3 — Description of Additional Supplementary File [file 42003_2025_9249_MOESM3_ESM.pdf]

Description of Additional Supplementary Files

File name: Supplementary Data 1

Description: The source data behind the graphs in the paper.
