# Supplementary material for: A GPER-PKA-Centrin axis regulates centrosome numbers and centriole integrity in colon cancer cells
Source: Commun Biol. 2025 Nov 26;8:1739. doi: 10.1038/s42003-025-09249-4 (PMC12673129; doi:10.1038/s42003-025-09249-4)
Supplement: Supplementary file 5 — Reporting Summary [file 42003_2025_9249_MOESM5_ESM.pdf]

nature portfolio

Corresponding author(s): Ailine Stolz

Last updated by author(s): Nov 3, 2025

Reporting Summary

Nature Portfolio wishes to improve the reproducibility of the work that we publish. This form provides structure for consistency and transparency in reporting. For further information on Nature Portfolio policies, see our Editorial Policies and the Editorial Policy Checklist.

Statistics

For all statistical analyses, confirm that the following items are present in the figure legend, table legend, main text, or Methods section.

n/a	Confirmed	
☐	☒	The exact sample size (*n*) for each experimental group/condition, given as a discrete number and unit of measurement
☐	☒	A statement on whether measurements were taken from distinct samples or whether the same sample was measured repeatedly
☐	☒	The statistical test(s) used AND whether they are one- or two-sided<br>*Only common tests should be described solely by name; describe more complex techniques in the Methods section.*
☒	☐	A description of all covariates tested
☒	☐	A description of any assumptions or corrections, such as tests of normality and adjustment for multiple comparisons
☐	☒	A full description of the statistical parameters including central tendency (e.g. means) or other basic estimates (e.g. regression coefficient) AND variation (e.g. standard deviation) or associated estimates of uncertainty (e.g. confidence intervals)
☐	☒	For null hypothesis testing, the test statistic (e.g. *F*, *t*, *r*) with confidence intervals, effect sizes, degrees of freedom and *P* value noted<br>*Give P values as exact values whenever suitable.*
☒	☐	For Bayesian analysis, information on the choice of priors and Markov chain Monte Carlo settings
☒	☐	For hierarchical and complex designs, identification of the appropriate level for tests and full reporting of outcomes
☒	☐	Estimates of effect sizes (e.g. Cohen's *d*, Pearson's *r*), indicating how they were calculated

Our web collection on statistics for biologists contains articles on many of the points above.

Software and code

Policy information about availability of computer code

Data collection

Immunofluorescence images were collected at a Zeiss AxioObserver Z1 microscope (Zeiss) equipped with an apotome 2.0 module, a heated chamber, and an AxioCam MRm camera (Zeiss) using the integrated ZEN 3.1 blue edition software (Carl Zeiss Microscopy GmbH).

TEM images were collected at a Jeol TEM the JEM 1400 Plus equipped with an lympus camera using the ITEM software (Olympus, Version ITEM-E-23082007).

Proteins from Western blot were documented with the Fusion Solo S.

Cell proliferation was determind by manual counting using a hemacytometer (purchased from Fein-OPTIK).

All graphs were created with PRISM 10.1.2. Figures were generated using Illustrator, version 27.8 (Adobe, San Jose, CA, USA).

Data analysis	Immunofluorescence images were analyzed using the ZEN 3.1 blue edition software (Carl Zeiss Microscopy GmbH). TEM images were analyzed using the TEM software (Olympus, Version ITEM-E-23082007).<br><br>Western blot bands were quantified using ImageJ (version 1.53a).<br><br>Statistics analysis were calculated with R.4.3.2 or R.4.0.2 and PRISM v.10.1.2 as indicated in the manuscript.<br><br>Immunofluorescence staining images and Western blot images were processed using ImageJ and Adobe Photoshop (version 25.7.0).

For manuscripts utilizing custom algorithms or software that are central to the research but not yet described in published literature, software must be made available to editors and reviewers. We strongly encourage code deposition in a community repository (e.g. GitHub). See the Nature Portfolio guidelines for submitting code & software for further information.

Data

Policy information about availability of data

All manuscripts must include a data availability statement. This statement should provide the following information, where applicable:

- Accession codes, unique identifiers, or web links for publicly available datasets
- A description of any restrictions on data availability
- For clinical datasets or third party data, please ensure that the statement adheres to our policy

All data generated in this study are provided in this article file, Supplementary Information, and Supplementary Data. This study includes no data deposited in external repositories. The relevant source data from each figure and the uncropped images of the immunoblots presented in this study are included in Supplementary Data 1 or provided as a Supplementary Fig.

Research involving human participants, their data, or biological material

Policy information about studies with human participants or human data. See also policy information about sex, gender (identity/presentation), and sexual orientation and race, ethnicity and racism.

Reporting on sex and gender	Human colorectal cancer cell line HCT116 (male) and human colorectal cancer cell line HCT-15 (male).
Reporting on race, ethnicity, or other socially relevant groupings	N/A
Population characteristics	N/A
Recruitment	N/A
Ethics oversight	N/A

Note that full information on the approval of the study protocol must also be provided in the manuscript.

Field-specific reporting

Please select the one below that is the best fit for your research. If you are not sure, read the appropriate sections before making your selection.

☒ Life sciences ☐ Behavioural & social sciences ☐ Ecological, evolutionary & environmental sciences

For a reference copy of the document with all sections, see nature.com/documents/nr-reporting-summary-flat.pdf

Life sciences study design

All studies must disclose on these points even when the disclosure is negative.

Sample size	For all quantitative data described in the article, the sample size (n) was reported as an exact number in the corresponding figure legends. Sample sizes to determine centrosome amplication upon different conditions are related to the publication from Bühler et al., 2022 (Bühler at al., Life Sci Alliance, 2022; doi: 10.26508/lsa.202201499). Initial pilot experiments for enlarged/P-Centrin were performed to estimate intra-variability of experimental conditions and to determine optimal sample size for statitical analysis.
Data exclusions	No data were excluded with exception for cAMP assays. Here, we set a cut-off derived from the respective positive control-treatment that was set to 50% for HCT116 or 25% for HCT-15 related to FSK (Fig. 1e, f) and 30% for HCT116 or 25% for HCT-15 related to G-1 (Fig. S1c). Values below these cut-offs were excluded and values above these cut-offs were considered as valid. The different cut-offs were choosen related to different base-levels of RLU values between both cell lines and treatments with the respective positive control. 1 experiment that was excluded for HCT116 (Fig. S1c) showed abnormally low RLU already upon DMSO-treatment compared to all other experiments, indicating a failure related to cell seeeding; and 1 experiment was excluded for HCT-15 (Fig. S1c) because the RLU values for DMSO-treatment were abnormally high compared to all other experiments, indicating a failure related to cell seeeding.
Replication	All quantifications are based at least on n = 3 biological replicates, with exception of TEM-analysis: n = 1 (DMSO) and n = 3 (BPA).

Replication	All quantifications of centrosome amplification are based on 3-4 independent experiments (biological replicates).<br>Determination of cAMP level and PKA activity is based on the evaluation of 5-6 (cAMP) or 6-7 (PKA) independent experiments (biological replicates). PKA assays were performed in two technical replicates. Proximity Ligation Assay is performed in two biological replicates and representative images are provieded in the manuscript.<br>Quantifications of the relative protein levels for western blot shown in Supplementary Fig. 7a were based on 3 different experiments (biological replicates).<br>Quantification of signal intensities for Centrin-pS170 normalized to total Centrin-2 and for PKA normalized to g-tubulin signals was based on 3 different experiments (biological replicates).<br>The proportion of centrosome amplified cells with enlarged or phosphorylated Centrin-2 foci was determined from 3-4 different experiments (biological replicates).<br>To quantify enlarged P-Centrin, the same immunofluorescence slides used to assess the proportion of enlarged Centrin were simultaneously analyzed for P-Centrin.<br>Cell proliferation assays were performed in three or four different experiments (biological replicates).<br>Representative examples of immunofluorescence experiments that are shown in the figures were repeated at least 3 times. Proximity Ligation Assay was repeated 2 times.<br>Representative examples of western blots that are shown in the figures were repeated at least 3 times.
Randomization	This in vitro study is based on the analyses of single cells (centrosome amplification; enlarged and p-centrin), whole cell populations (cell proliferation) and whole cell extracts (Western blot and PKA/cAMP assays), and randomization did not apply. As described in the Results and Materials and Methods sections, cells were seeded and then treated with the different substances for the indicated time periods.
Blinding	The investigators who collected these data were not blinded to the substance treatments and the analysis of centrosome amplification and the proportion of cells with enlarged or phosphorylated Centrin. Unbiased analysis of data was carried out wherever possible.<br><br>All scientists were trained to recognize the relevant phenotypes. The last author also randomly re-quantified some experiments to ensure accuracy. Two independent scientists quantified centrosome amplification and enlarged/p-Centrin phenotypes, producing consistent results. These quantifications were repeated using the same treatments (E2, BPA. DES, ICI, Tam, G-1) across multiple (sub-)figures and verified in two different cell lines, both yielding consistent outcomes. To analyze centrosome amplification and enlarged/p-Centrin, various substances, including endogenous estrogens, xenoestrogens, and synthetic ligands, were used, all producing consistent effects. Additionally, two different Centrin-antibodies were employed to quantify centrosome amplification and enlarged Centrin-labeled centrioles, as described in the results section. Quantitative data for cAMP and PKA assays were automatically analyzed using the Synergy Neo2 Reader (BioTek) or the TECAN Infinite M200 plate reader.

Reporting for specific materials, systems and methods

We require information from authors about some types of materials, experimental systems and methods used in many studies. Here, indicate whether each material, system or method listed is relevant to your study. If you are not sure if a list item applies to your research, read the appropriate section before selecting a response.

Materials & experimental systems

n/a	Involved in the study	
☐	☒	Antibodies
☐	☒	Eukaryotic cell lines
☒	☐	Palaeontology and archaeology
☒	☐	Animals and other organisms
☒	☐	Clinical data
☒	☐	Dual use research of concern
☒	☐	Plants

Methods

n/a	Involved in the study	
☒	☐	ChIP-seq
☒	☐	Flow cytometry
☒	☐	MRI-based neuroimaging

Antibodies

Antibodies used	Antibodies used in this article are described in the Material and Methods section.<br><br>Primary antibodies for western blot and dilutions used were as follows:<br>mouse anti-flag (WB 1:700, clone M2, F3165, Millipore Sigma);<br>mouse anti-α-tubulin (WB 1:2000, clone B-5-1-2, sc-23948, Santa Cruz);<br>mouse anti-actin (WB 1:60,000, clone AC-15, F3022, Sigma);<br>mouse anti-HSP90 alpha/beta (WB 1:500, clone F-8, sc-13119, Santa Cruz);<br>mouse anti-PKA Cα (WB 1:500, clone A-2, sc-28315, Santa Cruz);<br>rabbit anti-PKA Cα (WB 1:1000, 4782, Cell Signaling);<br>mouse anti-PKA IIα reg (WB 1:500, H-12, sc-137220, Santa Cruz);<br>rabbit anti-PKA IIα reg (WB 1:500, # A301-670A, ThermoFisher);<br>rabbit anti-AKAP-9 (WB 1:500, ab237752, Abcam);<br>rabbit anti-Centrin-2 (WB 1:1000, ABE480, Merk Millipore);<br>rabbit anti-phospho-Ser170-centrin-2 (WB 1:430, 7TM Antibodies GmbH);<br>mouse anti-Vinculin (WB 1:2000, sc-73614, Santa Cruz);<br>rabbit anti-Plk4 (WB 1:1000, 12952-1-AP, Proteintech).<br><br>Secondary anti-rabbit or anti-mouse antibodies conjugated to HRP or fluorophores were used at 1:10000 (111-035-146,

111-035-144, Jackson Immuno-Research, or 926-32210, 926-68071, Licor).

Primary antibodies for immunofluorescences and dilutions used were as follows:
rabbit anti-phospho-Ser170-centrin-2 (IF 1:25, 7TM Antibodies GmbH);
rabbit anti-g-tubulin (IF 1:650, T3559, Sigma);
mouse anti-α-tubulin (IF 1:650, clone B-5-1-2, sc-23948, Santa Cruz);
mouse anti-Centrin-2/3 (IF 1:300, clone 20H5, 04-1624, Sigma);
rat anti-Centrin-2 (IF/ExM 1:150, clone W16110A, 698602, BioLegend);
mouse anti-Centrobin (IF 1:1000, ab70448, abcam);
rabbit anti-Cep135 (IF 1:300, ab75005, Abcam);
rabbit anti-CP110 (IF 1:100, ab243696, Abcam);
mouse anti-gamma-tubulin (IF 1:650/Exm 1.500, clone GTU88, T6557, Sigma);
Alexa Fluor® 647 anti-gamma tubulin (IF 1:50, clone TU-30 , ab191114), abcam)

Secondary antibodies conjugated to Alexa Fluor-488/-555 (1:1000, A-11029, A-21428, Invitrogen)
ExM: Secondary antibodies conjugated to Alexa Fluor 488 or 555 (1:500, A-11006, A-21424, Invitrogen)

Validation

Antibodies from different companies were validated by Western Blot and /or immunofluorescences for sensitivity and specificity following the manufacture indications and suggestions. Antibodies were chosen based on the literature (see Refs) and were tested with positive and negative controls where possible before the experiment was done.

mouse anti-flag (WB 1:700, clone M2, F3165, Millipore Sigma) is a tested mouse monoclonal antibody and recognizes the FLAG sequence of FLAG fusion proteins at the N-terminus, Met-N-terminus, and C-terminus. The antibody can also detect FLAG at internal sites.; purified immunoglobulin (Purified IgG1 subclass) using Protein A. (Bühler at al., Life Sci Alliance, 2022; doi: 10.26508/lsa.202201499)

mouse anti-α-tubulin (WB 1:2000, clone B-5-1-2, sc-23948, Santa Cruz) is a tested mouse monoclonal antibody raised against Sarkosyl-resistant ribbons from Strongylocentrotus purpuratus (Sea Urchin) sperm axonemes. (Bühler at al., Life Sci Alliance, 2022; doi: 10.26508/lsa.202201499)

mouse anti-actin (WB 1:60,000, clone AC-15, F3022, Sigma) is a tested mouse monoclonal antibody, FITC conjugate, and raised against slightly modified β-cytoplasmic N-terminal actin peptide, Ac-Asp-Asp-Asp-Ile-Ala-Ala-Leu-Val-Ile-Asp-Asn-Gly-Ser-Gly-Lys, conjugated with KLH.; purified immunoglobulin. (Bühler at al., Life Sci Alliance, 2022; doi: 10.26508/lsa.202201499)

mouse anti-HSP90 alpha/beta (WB 1:500, clone F-8, sc-13119, Santa Cruz) is a tested mouse monoclonal antibody raised against the amino acid sequence 610-723 of HSP 90β from the human species. (Li et al., Andrology, 2021; doi: 10.1111/andr.12862)

mouse anti-PKA Cα (WB 1:500, clone A-2, sc-28315, Santa Cruz) is a tested mouse monoclonal antibody, raised against cAMP-dependent protein kinase catalytic subunit alpha (Human). UniProt P17612 - KAPCA_HUMAN

rabbit anti-PKA Cα (WB 1:1000, 4782, Cell Signaling) is a tested rabbit polyclonal antibody, produced by immunizing animals with a synthetic peptide corresponding to the carboxy terminal sequence of human PKA C-α. Antibodies are purified by protein A and peptide affinity chromatography. The antibody detects endogenous levels of total PKA C-α. UniProt ID: #P17612

mouse anti-PKA IIα reg (WB 1:500, H-12, sc-137220, Santa Cruz) is a tested mouse monoclonal antibody raised against amino acids 21-100 deletion 39-54 (deletion 39-54) mapping near the N-terminus of PKA IIα reg of human origin.

rabbit anti-PKA IIα reg (WB 1:500, # A301-670A, ThermoFisher) is a tested rabbit polyclonal antibody raised against a region between residue 50 and 100 of Human protein kinase, cAMP-dependent, regulatory, type II, alpha.

rabbit anti-AKAP-9 (WB 1:500, ab237752, Abcam) is a tested rabbit polyclonal antibody and reacts with Recombinant fragment - Human, Human samples. Immunogen corresponding to Recombinant Fragment Protein within Human AKAP9 aa 50-200. The exact immunogen used to generate this antibody is proprietary information. Database link: Q99996

rabbit anti-Centrin-2 (WB 1:1000, ABE480, Merk Millipore) is a tested rabbit polyclonal antibody, raised against human Centrin-2. Immunogen is a recombinant protein corresponding to human Centrin-2. UniProt ID: P41208

rabbit anti-phospho-Ser170-centrin-2 (WB 1:430, 7TM Antibodies GmbH) antibody is provided by 7TM Antibodies GmbH. Three rabbits were immunized by using a synthetic phosphopeptide spanning the region around S170 in the human Centrin-2 sequence [Cys-LRIMKKT(pS)LY172]. The peptides were coupled to keyhole limpet hemocyanin (KLH). Antisera were used for affinity purification against their immunizing peptides, and specificity was then verified with synthetic phosphopeptides and corresponding non-phosphopeptides using dot-blot assays. The antibody was further tested/characterized within this study (Fahrländer et al.).
mouse anti-Vinculin (WB 1:2000, clone 7F9 sc-73614, Santa Cruz) is a tested mouse monoclonal antibody raised against vinculin of human origin.

rabbit anti-Plk4 (WB 1:1000, 12952-1-AP, Proteintech) is a tested rabbit polyclonal antibody, that recognizes human polo like kinase 4. Immunogen corresponding to PLK4 fusion protein Ag3605.

rabbit anti-gamma-tubulin (IF 1:650, T3559, Sigma) is a tested rabbit polyclonal antibody, that recognizes an epitope located in the N-terminal region of gamma-tubulin (amino acids 38-53).; IgG fraction of antiserum. UniProt ID: P23258

mouse anti-gamma-tubulin (IF 1:650/Exm 1.500, clone GTU88, T6557, Sigma) is a tested mouse monoclonal antibody recognizes an epitope located in the N-terminal amino acids of γ-tubulin (48 kDa). Immunogen corresponding to synthetic γ-tubulin peptide conjugated to KLH. UniProt ID: P23258

Alexa Fluor® 647 anti-gamma tubulin (IF 1:50, clone TU-30 , ab191114, abcam) is a tested mouse monoclonal TBG1 antibody -

conjugated to Alexa Fluor® 647. C-terminal. Immunogen corresponding to Synthetic Peptide within Human TUBG1 aa 400-450 conjugated to Keyhole Limpet Haemocyanin. The exact immunogen used to generate this antibody is proprietary information. Database link: P23258.

mouse anti-Centrin-2/3 (IF 1:300, clone 20H5, 04-1624, Sigma) is a tested mouse monoclonal antibody. The immunogen is a recombinant protein corresponding to the C-terminus of Chlamydomonas Centrin. UniProt ID: Q12798

rat anti-Centrin-2 (IF/ExM 1:150, clone W16110A, 698602, BioLegend) is a tested rat monoclonal antibody raised against 172 amino acids with a predicted molecular weight of 19.7 kD. Contains four EF-hand domains.

mouse anti-Centrobin (IF 1:1000, ab70448, abcam) is a tested mouse polyclonal antibody. Immunogen corresponding to recombinant full length protein corresponding to Human CNTROB.

rabbit anti-Cep135 (IF 1:300, ab75005, Abcam) is a tested rabbit polyclonal antibody. Immunogen corresponding to Synthetic Peptide within Human CEP135. The exact immunogen used to generate this antibody is proprietary information. Database link: Q66GS9.

rabbit anti-CP110 (IF 1:100, ab243696, Abcam) is a tested rabbit polyclonal antibody. Immunogen corresponding to Recombinant Fragment Protein within Human CCP110 aa 250-450. The exact immunogen used to generate this antibody is proprietary information. Database link: O43303.

Eukaryotic cell lines

Policy information about cell lines and Sex and Gender in Research

Cell line source(s)

HCT15 (male) was purchased from the American Type Culture Collection (ATCC); ATCC® CCL225™
HCT116 (male) were purchased from the German Collection of Microorganisms and Cell Cultures GmbH (DSMZ).

Authentication

The identities of all cell lines were verified using the Eurofins Genomics Cell Line Authentication service (Eurofins Genomics, Ebersberg, Germany).

Mycoplasma contamination

The cell lines were routinely checked for mycoplasma contamination tested using the Eurofins Genomics mycoplasma test service (Eurofins Genomics, Ebersberg, Germany). All cell lines used were tested negative.

Commonly misidentified lines
(See ICLAC register)

None

Plants

Seed stocks

N/A

Novel plant genotypes

N/A

Authentication

N/A
